# Posturographic Evidence of Sensory Integration Deficits and Postural Instability in Alzheimer’s Disease

**DOI:** 10.1002/alz70861_108344

**Published:** 2025-12-23

**Authors:** Michele da Rocha Anselmo, Lucélia Epifânio Pereira da Silva, Bianca Melo Gonçalves, Lara Catarine Inácio da Silva, Maria Aparecida Camargos Bicalho, Ludimila Labanca

**Affiliations:** ^1^ Postgraduate Program in Speech‐Language Pathology Sciences, Belo Horizonte, Belo Horizonte Brazil; ^2^ Universidade Federal de Minas Gerais, Belo Horizonte, Minas Gerais Brazil; ^3^ Cog‐Aging Research Group, Belo Horizonte, Minas Gerais Brazil; ^4^ Postgraduate Program in Speech‐Language Pathology Sciences, Belo Horizonte, Minas Gerais Brazil; ^5^ Jenny de Andrade Faria Institute – Outpatient Reference Center for the Elderly, Universidade Federal de Minas Gerais (UFMG), Belo Horizonte, Minas Gerais Brazil; ^6^ Department of Clinical Medicine, Faculty of Medicine, Universidade Federal de Minas Gerais (UFMG), Belo Horizonte, Minas Gerais Brazil; ^7^ Geriatrics and Gerontology Center Clinical Hospital of Universidade Federal de Minas Gerais, Belo Horizonte, Minas Gerais Brazil; ^8^ Cog‐Aging Research Group, Universidade Federal de Minas Gerais (UFMG), Belo Horizonte, Minas Gerais Brazil; ^9^ Older Adult’s Psychiatry and Psychology Extension Program (PROEPSI), School of Medicine, Universidade Federal de Minas Gerais (UFMG), Belo Horizonte, Minas Gerais Brazil

## Abstract

**Background:**

Falls are one of the leading causes of mortality and morbidity among older adults worldwide. Approximately 26.5% of elderly individuals experience at least one fall within a one‐year period, a percentage that rises to 44.27% among those diagnosed with Alzheimer's Disease (AD). AD is a progressive neurodegenerative disease characterized by the abnormal accumulation of beta‐amyloid and tau proteins, accounting for 70% of dementia cases globally. Postural balance is frequently impaired in AD, increasing the risk of falls and their consequences. Therefore, it is important to understand the sensory factors related to these concerning numbers in order to develop effective prevention policies.

**Method:**

This is an analytical cross‐sectional study involving 45 older adults divided into two groups matched by age and sex: 25 with AD and 20 controls. Clinical and cognitive characterization was carried out through medical and neuropsychological assessment, and the diagnosis of AD was confirmed by anatomopathological examination. Static posturography with dynamic tests was conducted using the Horus® force platform to measure the center of pressure (CoP) and its oscillation under different sensory conditions. The limits of stability, which represent the ability to shift the CoP without losing balance, were assessed, along with sensory integration tests that analyze the integration of visual, vestibular, and somatosensory systems in postural control. Statistical analysis was performed using the chi‐square and Mann‐Whitney tests, with *p* <0.05 considered significant.

**Result:**

The groups did not differ in terms of age, sex, weight, and education level. In the posturography tests, the AD group showed poorer performance in the limits of stability (*p* <0.032), overall balance index (*p* =0.002), visual index (*p* =0.002), vestibular index (*p* <0.002), and visual dependency (*p* =0.014), with no significant difference in the somatosensory index (*p* =0.553).

**Conclusion:**

The findings indicate that older adults with AD exhibit significant deficits in postural control, particularly under conditions requiring visual and vestibular integration, which may contribute to the increased risk of falls in this population.

